# Supplementary material for: An evolutionary hotspot defines functional differences between CRYPTOCHROMES
Source: Nat Commun. 2018 Mar 19;9:1138. doi: 10.1038/s41467-018-03503-6 (PMC5859286; doi:10.1038/s41467-018-03503-6)
Supplement: Supplementary file 1 — Supplementary Information(PDF 7767 kb) [file 41467_2018_3503_MOESM1_ESM.pdf]

Supplementary Information

An evolutionary hotspot defines functional differences between CRYPTOCHROMES

Rosensweig et al.

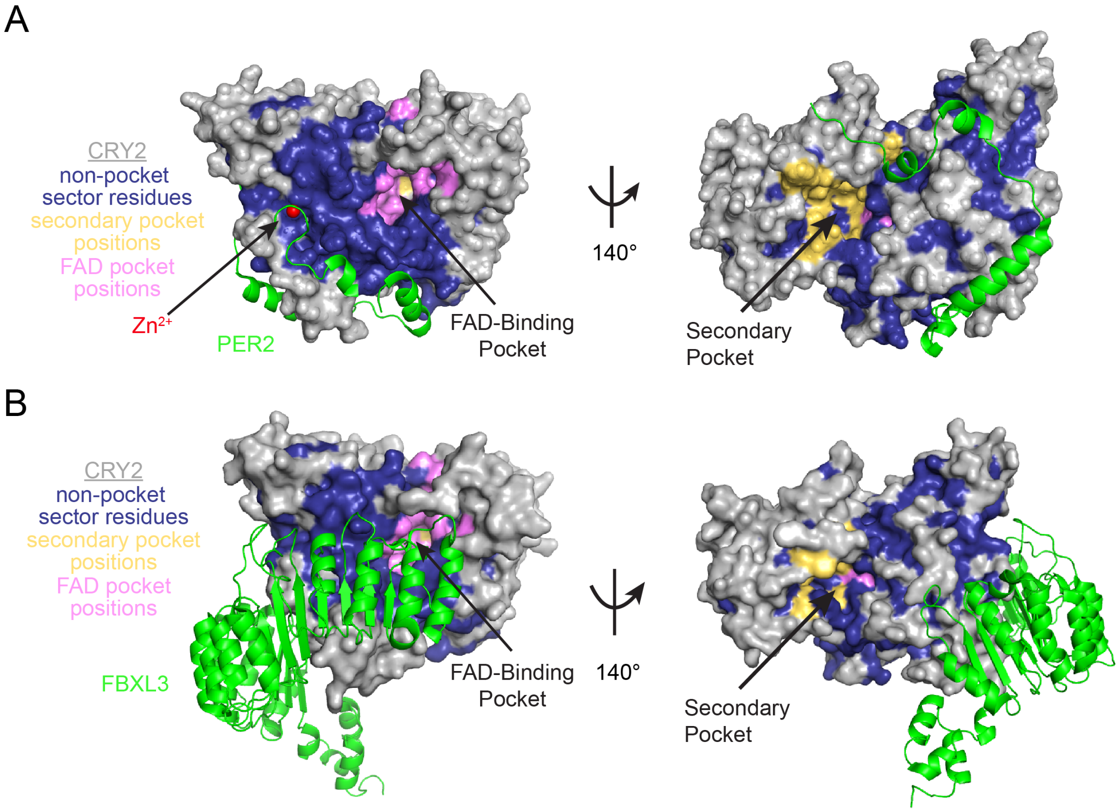

Supplementary Figure 1. Sector positions surrounding the FAD binding pocket interact with PER2 and FBXL3. (A) Shows two views of the CRY2/PER2 CRY-binding domain (CBD) complex (PDB: 4U8H). The PER2 CBD is shown as a green cartoon helix and CRY2 is colored as in Fig. 1d to show the sector residues and secondary and FAD pocket residues from the sector. The figure on the left depicts the FAD-binding pocket and the CC helix of CRY2 while the figure on the right is rotated to show the side of CRY2 containing the secondary pocket. PER2 interacts heavily with residues forming the SCA network. (B) Two views of the FBXL3/CRY2 complex (PDB: 4I6J) with FBXL3 in green and CRY2 colored as in Fig. 1d. The view at the left shows FBXL3's C-terminal tail penetrating into CRY2's FAD-binding pocket, demonstrating the degree to which this critical interaction depends on SCA network residues. The view on the right shows a rotated CRY2, depicting the secondary pocket and FBXL3's embrace of the CC helix of CRY2 and its interaction with SCA network residues.

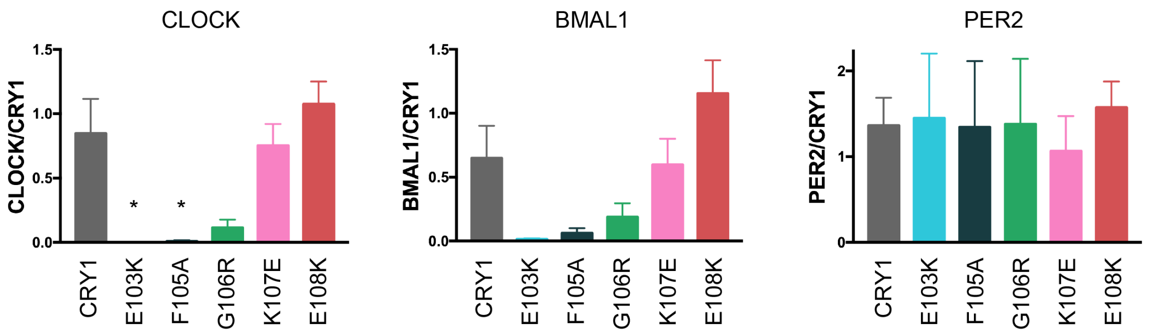

Supplementary Figure 2. Quantification of co-immunoprecipitation experiments in Fig. 3d. Graphs show respectively the ratio of CLOCK, BMAL1, and PER2 to CRY1 in the IP blot from three independent experiments. Quantification was performed by densitometric analysis with ImageJ. Asterisks show significance of the CLOCK/CRY ratio compared to WT by unpaired t-test with Welch's correction (E103K, *, $p = 0.0346$ and F105A, *, $p = 0.0357$).

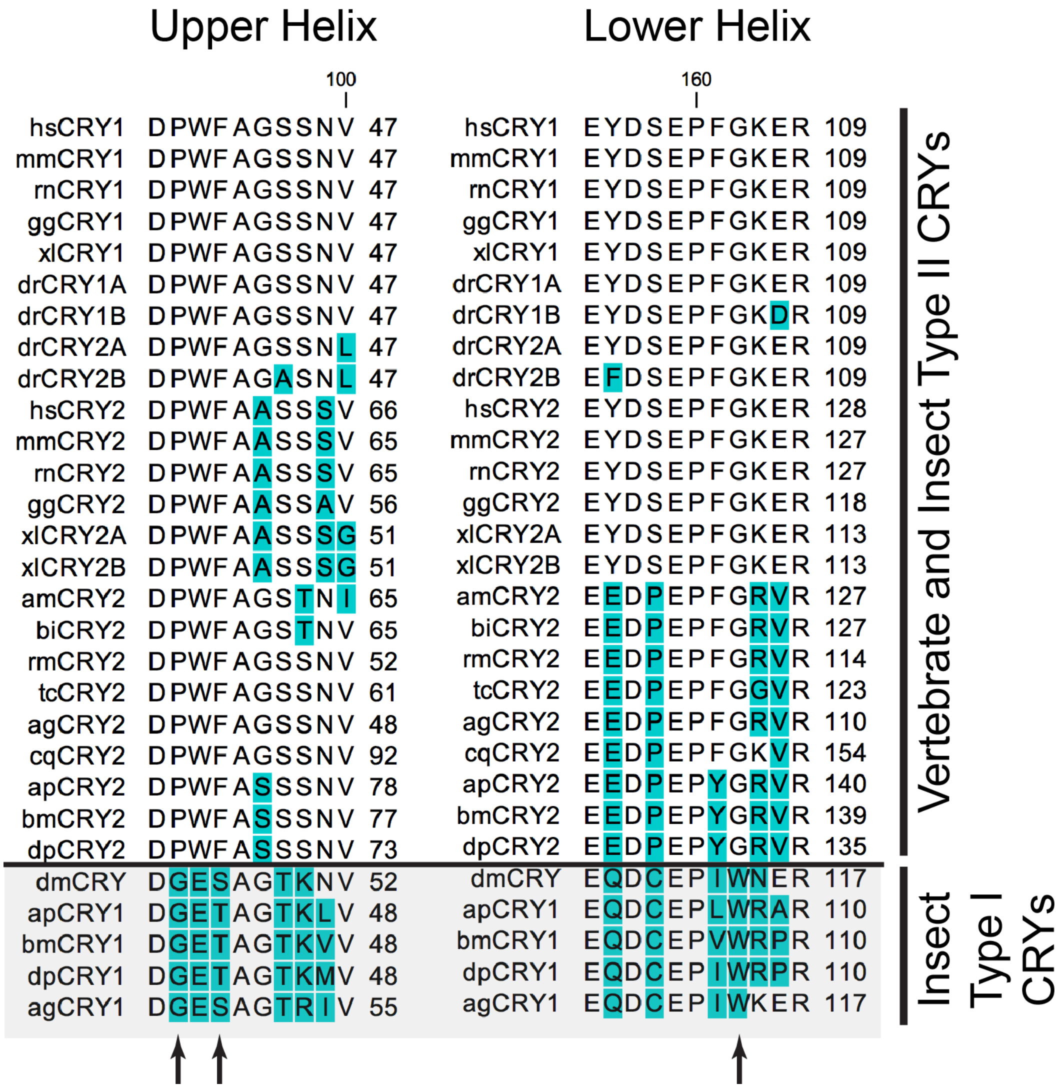

Supplementary Figure 3. Conserved structural features of vertebrate-like CRYs. Shows an alignment of the upper and lower boundaries of the secondary pocket from type I and type II CRYs. Position within the alignment is indicated by the number at the top and position within each primary sequence is indicated by the number at the right of each sequence. Arrows at the bottom indicate residues that are highly conserved within each group and divergent between the two groups, further explored in Figure 4. Green shading indicates divergence from consensus sequence. Abbreviations: hs (*Homo sapiens*, human), mm (*Mus musculus*, mouse), rn (*Rattus norvegicus*, rat), gg (*Gallus gallus*, chicken), xl (*Xenopus laevis*, African clawed frog), dr (*Danio rerio*, zebrafish), am (*Apis mellifera*, western honey bee), bi (*Bombus impatiens*, eastern bumblebee), rm (*Rhyparobia maderae*, madeira cockroach), tc (*Tribolium castaneum*, red flour beetle), ag (*Anopheles gambiae*, marsh mosquito), cq (*Culex quinquefasciatus*, southern house mosquito), ap (*Antheraea pernyi*, Chinese tussar moth), bm (*Bombyx mori*, domesticated silk moth), dp (*Danaus plexippus*, monarch butterfly), dm (*Drosophila melanogaster*, fruit fly).

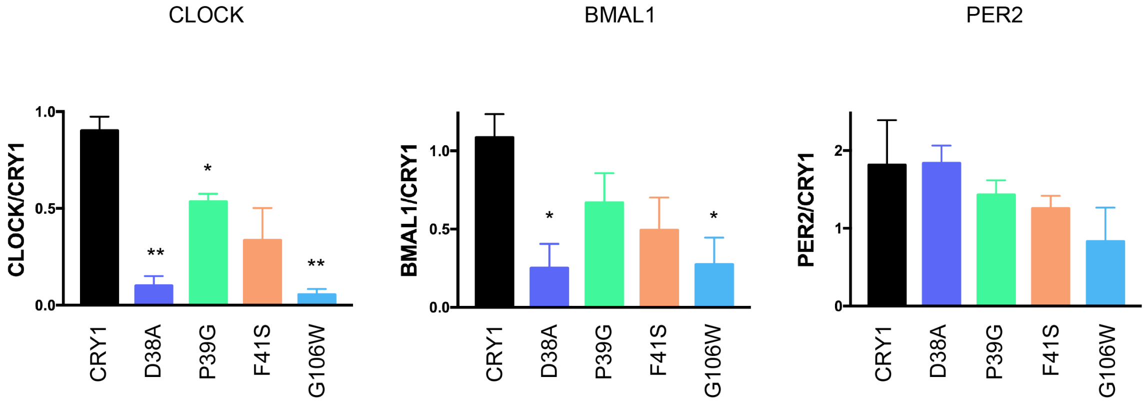

Supplementary Figure 4. Quantification of co-immunoprecipitation experiments in Fig. 4e. Graphs show respectively the ratio of CLOCK, BMAL1, and PER2 to CRY1 in the IP blot from three independent experiments. Quantification was performed by densitometric analysis with ImageJ. The CLOCK/CRY ratios of D38A (**, $p = 0.0014$), P39G (*, $p = 0.0198$), and F41s (**, $p = 0.0030$) were significantly different from WT by unpaired t-test with Welch's correction. The BMAL1/CRY ratios of D38A (*, $p = 0.0180$) and G106W (*, $p = 0.0245$) were significantly different from WT by unpaired t-test with Welch's correction.

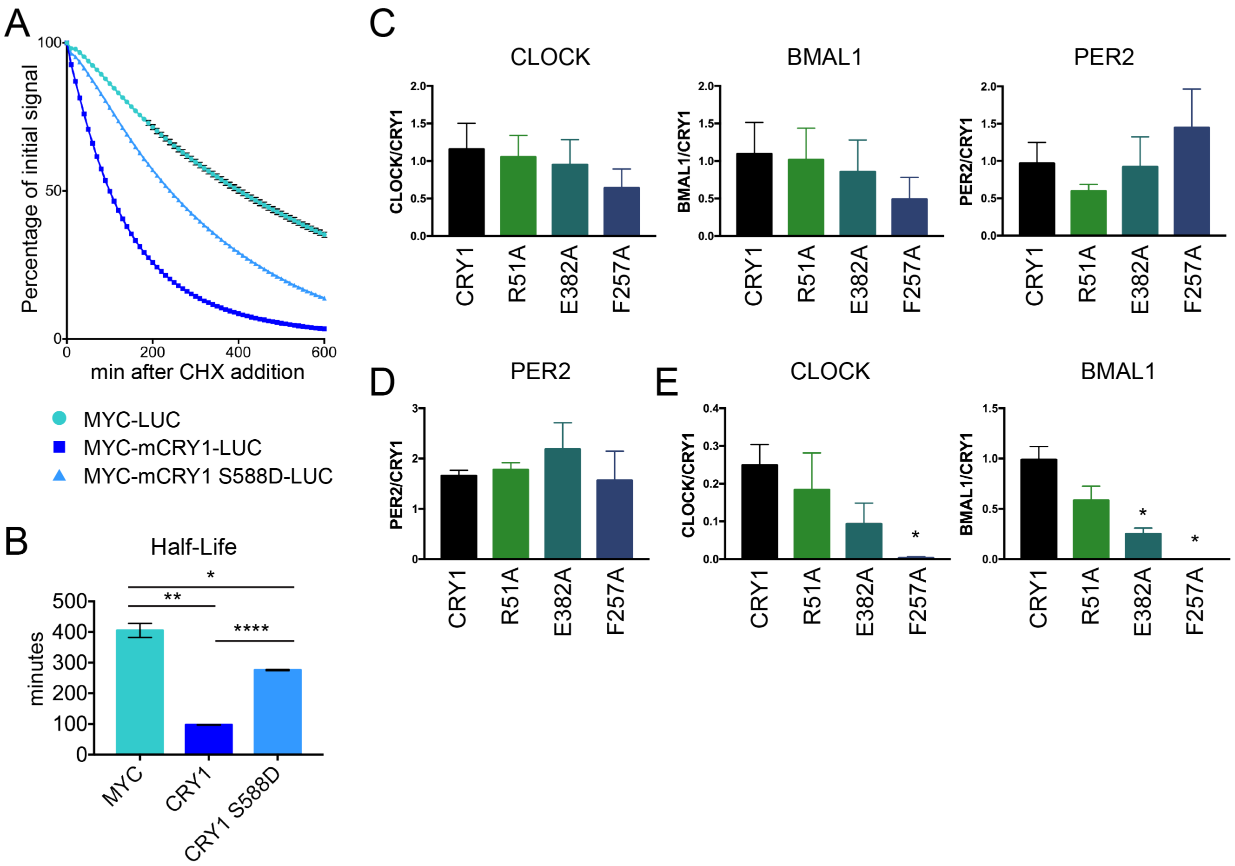

Supplementary Figure 5. Validation of CRY1::LUC fusion construct and quantification of co-immunoprecipitation experiments in Fig. 5. (A) A *Luc* gene was fused to the C-terminus of a *Myc-Cry1* expression vector. MYC-CRY1-LUC was constitutively expressed in 293A cells for 48 hours before treatment with cycloheximide and the decay in luminescence was monitored as a reporter for protein degradation. Shown here are the WT vector and two controls to demonstrate the efficacy of the approach (n = 3/condition). Samples were normalized to their initial luminescent signal and graphed as the decay from that initial signal. Shown as mean ± SEM. MYC-LUC is a fusion of the MYC tag to LUC. The CRY1 S588D mutation has been previously shown to stabilize CRY1 (Gao et al., 2013; Papp et al., 2015). (B) Half-lives are shown as mean ± SEM. Half-life was determined by fitting a one-phase decay curve to the data in (A). Asterisks show significance by unpaired t-test with Welch's correction (*, $p = 0.0297$, **, $p = 0.0055$, ****, $p < 0.0001$). (C) Graphs show respectively the ratio of CLOCK, BMAL1, and PER2 to CRY1 in the IP blots from three independent experiments in which all four proteins were expressed together. Quantification was performed by densitometric analysis with ImageJ for the graphs in (C, D, and E). (D) Shows the ratio of PER2 to CRY1 in the IP blots from three independent experiments in which the two proteins were expressed together. (E) Two graphs that show respectively the ratio of CLOCK and BMAL1 to CRY1 in the IP blots from three independent experiments in which the three proteins were expressed together. The CLOCK/CRY ratio of F257A (*, $p = 0.0464$) was

significantly different from WT by unpaired t-test with Welch's correction. The BMAL1/CRY ratios of E382A (*, $p = 0.0173$) and F257A (*, $p = 0.0168$) were significantly different from WT by unpaired t-test with Welch's correction.

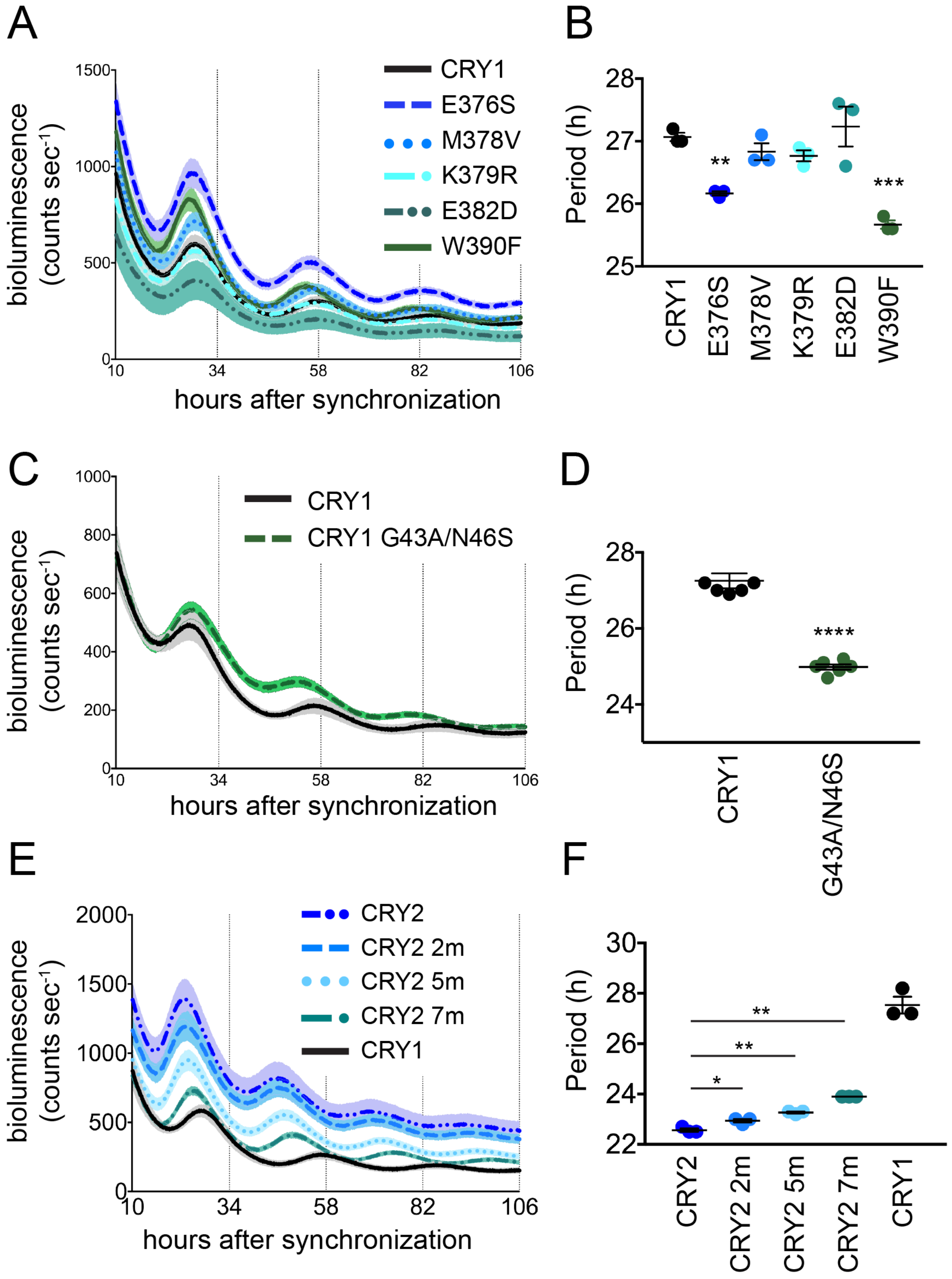
A
CRY1
E376S
M378V
K379R
E382D
W390F
bioluminescence (counts sec-1)
hours after synchronization
B
Period (h)
CRY1
E376S
M378V
K379R
E382D
W390F
C
CRY1
CRY1 G43A/N46S
bioluminescence (counts sec-1)
hours after synchronization
D
Period (h)
CRY1
G43A/N46S
E
CRY2
CRY2 2m
CRY2 5m
CRY2 7m
CRY1
bioluminescence (counts sec-1)
hours after synchronization
F
Period (h)
CRY2
CRY2 2m
CRY2 5m
CRY2 7m
CRY1

Supplementary Figure 6. Differences in CRY1 and CRY2 rescues are due to concerted effects of multiple mutations and distinct signaling responses. (A) Rescue assays performed with WT CRY1, and five single mutations of CRY1: E376S, M378V, K379R, E382D, W390F (n = 3/condition, representative of 9 (K379R, E382D), 12 (E376S, M378V), and 15 (W390F) plates from 3-5 independent experiments) shown as mean ± SEM. (B) Period plot of rescues in (A). Mean ± SEM indicated by bars. Asterisks show significance by unpaired t-test with Welch's correction (**, p = 0.0013, ***, p = 0.0001, ns (M378V: p = 0.2173; K379R: p = 0.0576; E382D: p = 0.6553)). (C) Rescue assays performed with WT CRY1 and a double mutation of CRY1, G43A/N46S (n = 6/condition, representative of 12 plates from 4 independent experiments) shown as mean ± SEM. (D) Period plot of rescues in (C). Mean ± SEM indicated by bars. Asterisks show significance by unpaired t-test with Welch's correction (****, p < 0.0001). (E) Rescue assays performed with WT CRY1, WT CRY2, CRY2 2m (CRY2 A61S/S64N), CRY2 5m (CRY2 S394E/V396M/R397K/D400E/F408W), and CRY2 7m (CRY2 A61G/S64N/S394E/V396M/R397K/D400E/F408W) shown as means ± SEM (n = 3/condition, reflective of 3 (CRY2 2X), 6 (CRY2 5X), 24 (CRY2 7X), and 33 (CRY2) plates from 1, 2, 8, and 11 independent experiments respectively). (F) Period plot of rescues in (E). Mean ± SEM indicated by bars. Asterisks show significance by unpaired t-test with Welch's correction compared to WT CRY2 (*, p = 0.0177, **, p < 0.01 (CRY2 5m: p = 0.0028; CRY2 7m: p = 0.0025; CRY1: p = 0.0034)).

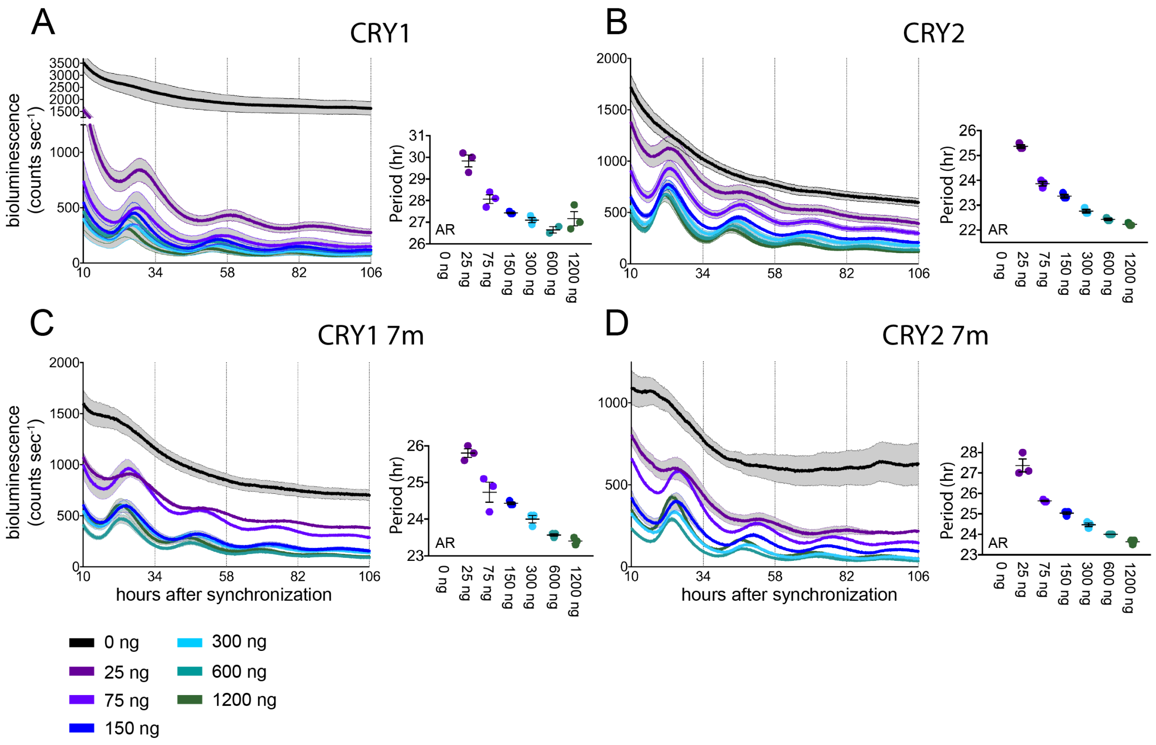

Supplementary Figure 7. Rescue vector dosage does not underlie period differences in *Cry1/Cry2* rescues. (A) Rescue assays performed with WT CRY1 (n = 3/dosage). *Cry1*$^{-/-}$/*Cry2*$^{-/-}$ MEFs were transfected with various amounts of rescue vector in a total of 5.2 µg of plasmid DNA. Mean ± SEM shown for each condition. The rescue period for each condition is shown in the graph to the right as mean ± SEM. (B) Rescue assays performed with WT CRY2 (n = 3/dosage). *Cry1*$^{-/-}$/*Cry2*$^{-/-}$ MEFs were transfected with various amounts of rescue vector in a total of 5.2 µg of plasmid DNA. Mean ± SEM shown for each condition. The rescue period for each condition is shown in the graph to the right as mean ± SEM. (C) Rescue assays performed with CRY1 7X (n = 3/dosage). *Cry1*$^{-/-}$/*Cry2*$^{-/-}$ MEFs were transfected with various amounts of rescue vector in a total of 5.2 µg of plasmid DNA. Mean ± SEM shown for each condition. The rescue period for each condition is shown in the graph to the right as mean ± SEM. (D) Rescue assays performed with CRY2 7X (n = 3/dosage). *Cry1*$^{-/-}$/*Cry2*$^{-/-}$ MEFs were transfected with various amounts of rescue vector in a total of 5.2 µg of plasmid DNA. Mean ± SEM shown for each condition. The rescue period for each condition is shown in the graph to the right as mean ± SEM.

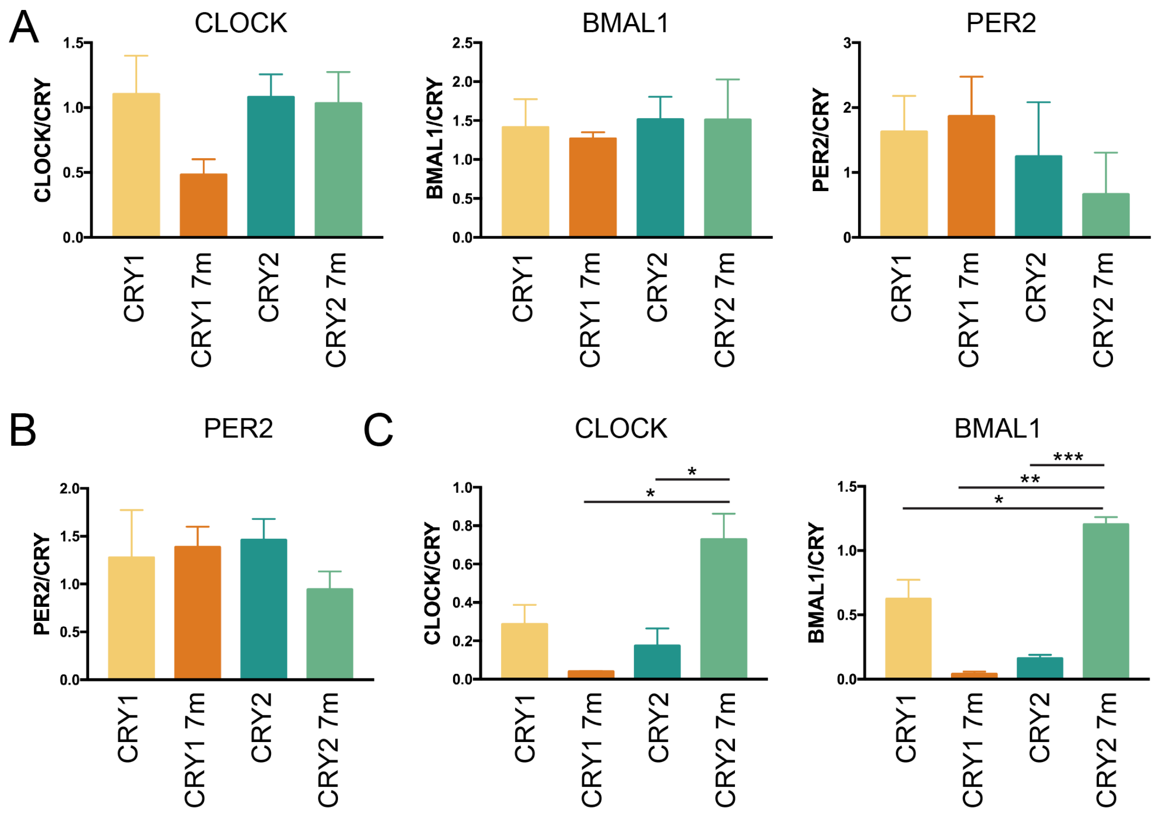

Supplementary Figure 8. Quantification of co-immunoprecipitation experiments from Fig. 7. (A) Graphs show respectively the ratio of CLOCK, BMAL1, and PER2 to CRY in the IP blot from three independent experiments (related to Fig. 7a). Quantification was performed by densitometric analysis with ImageJ. (B) Shows the ratio of PER2 to CRY1 in the IP blots from three independent experiments (related to Fig. 7b), quantified by densitometric analysis with ImageJ. (C) Two graphs that show respectively the ratio of CLOCK and BMAL1 to CRY1 in the IP blots from three independent experiments (related to Fig. 7c), quantified by densitometric analysis with ImageJ. The CLOCK/CRY ratio of CRY2 7m compared to CRY1 7m and CRY2 (*, $p = 0.0365$ and 0.0336 respectively) was significantly different by unpaired t-test with Welch's correction. The BMAL1/CRY ratio of CRY2 7m compared to CRY1, CRY1 7m, and CRY2 (*, $p = 0.0464$, **, $p = 0.0010$, ***, $p = 0.0005$) was significantly different by unpaired t-test with Welch's correction. The BMAL1/CRY ratio of CRY1 to CRY1 7m and CRY2 was not significant, but trending towards significance by unpaired t-test with Welch's correction ($p = 0.0576$ and 0.0846 respectively).

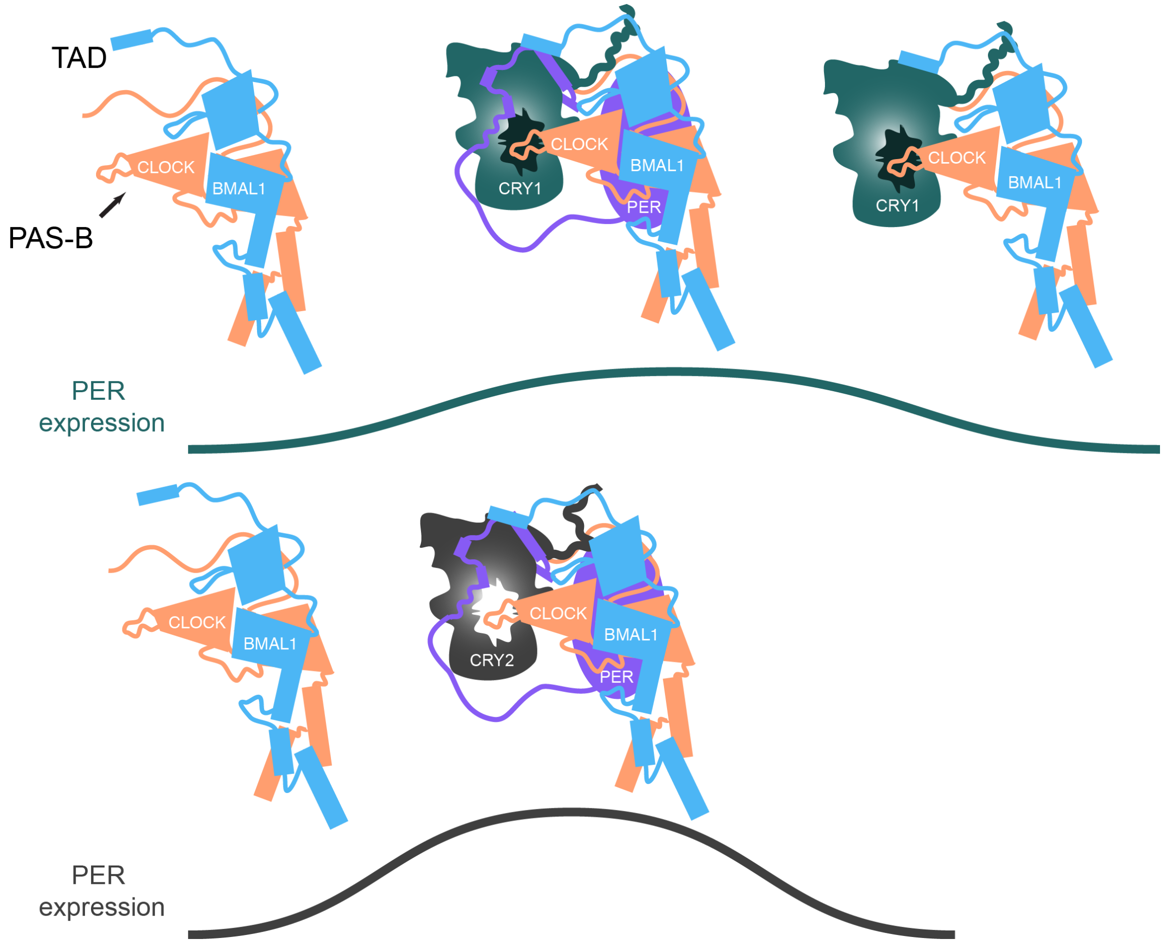

Supplementary Figure 9. Periodicity in the mammalian circadian clock depends on both the latent stability of the ternary complex and degradation dynamics. In the model shown here, cartoons are based on existing structures. During the rising phase of the oscillation, the BMAL1 TAD is free to recruit transcriptional components. As production of the repressors begins, CRY1 and CRY2 form a PER-dependent complex with CLOCK and BMAL1, allosterically interacting with BMAL1's TAD through C-terminal regions and with the CLOCK PAS B domain through the secondary pocket. Through these interactions, the activity of the CLOCK/BMAL1 heterodimer is suppressed. CRY2's repressive window is shortened due to its weakened ability to bind CLOCK and BMAL1 without PER. Later in the repressive phase, CRY1 is predominantly bound to CLOCK in a PER-independent interaction and sequesters the BMAL1 TAD. At all times, CRYs are subject to degradation primarily through interaction with FBXL3, eventually leading to renewal of the active phase of the clock.

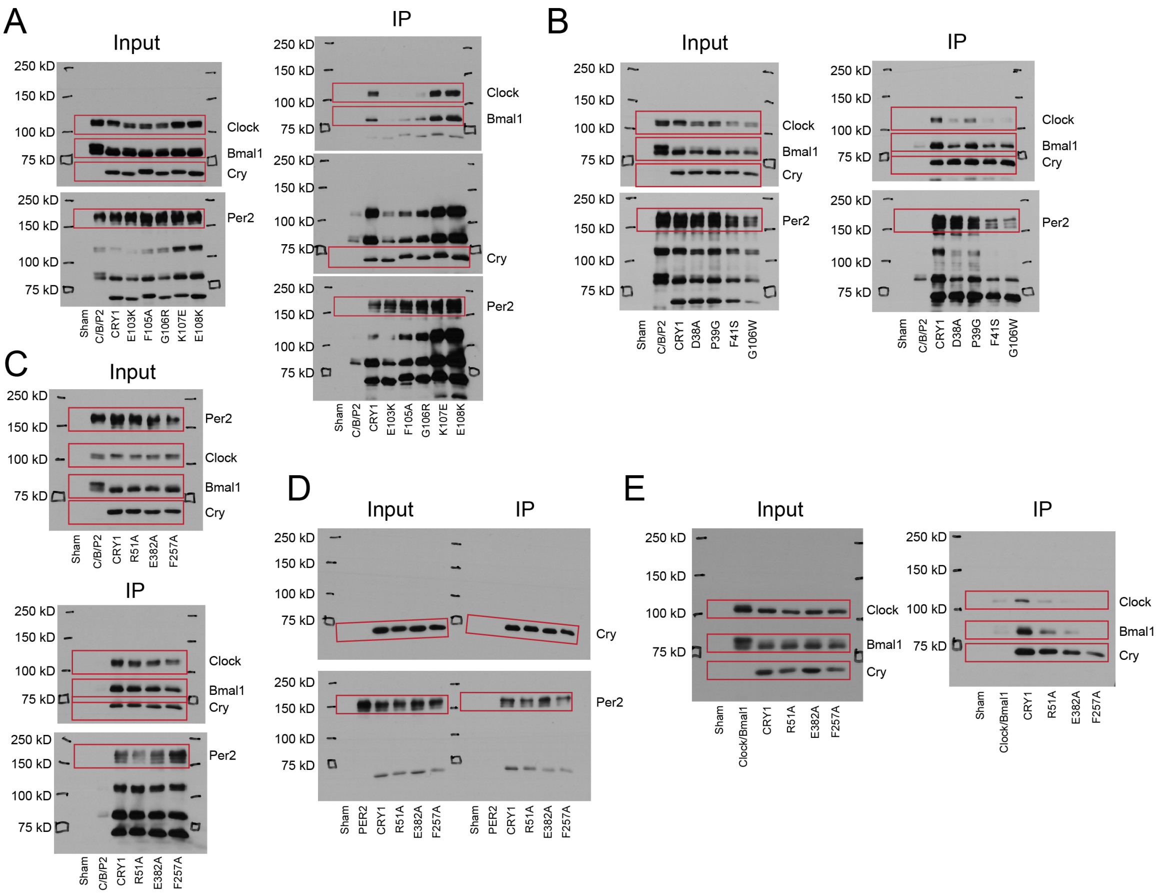

Supplementary Figure 10. Uncropped western blot images from Figures 3, 4, and 5. (A) At left are the uncropped western blot images used for the cropped input images in Figure 3. At right are the uncropped western blot images used for the cropped IP images in Figure 3. (B) At left are the uncropped western blot images used for the cropped input images in Figure 4. At right are the uncropped western blot images used for the cropped IP images in Figure 4. (C) At top is the uncropped western blot image used for the cropped input images in Figure 5g. On the bottom are the uncropped western blot images used for the cropped IP images in Figure 5g. (D) The left side of each image shows the uncropped western blot images used for the cropped input images in Figure 5h. The right side of each image shows the uncropped western blot images used for the cropped IP images in Figure 5h. (E) The left image shows the uncropped western blot image used for the cropped input images in Figure 5i and the right image shows the uncropped western blot image used for the cropped IP images in Figure 5i.

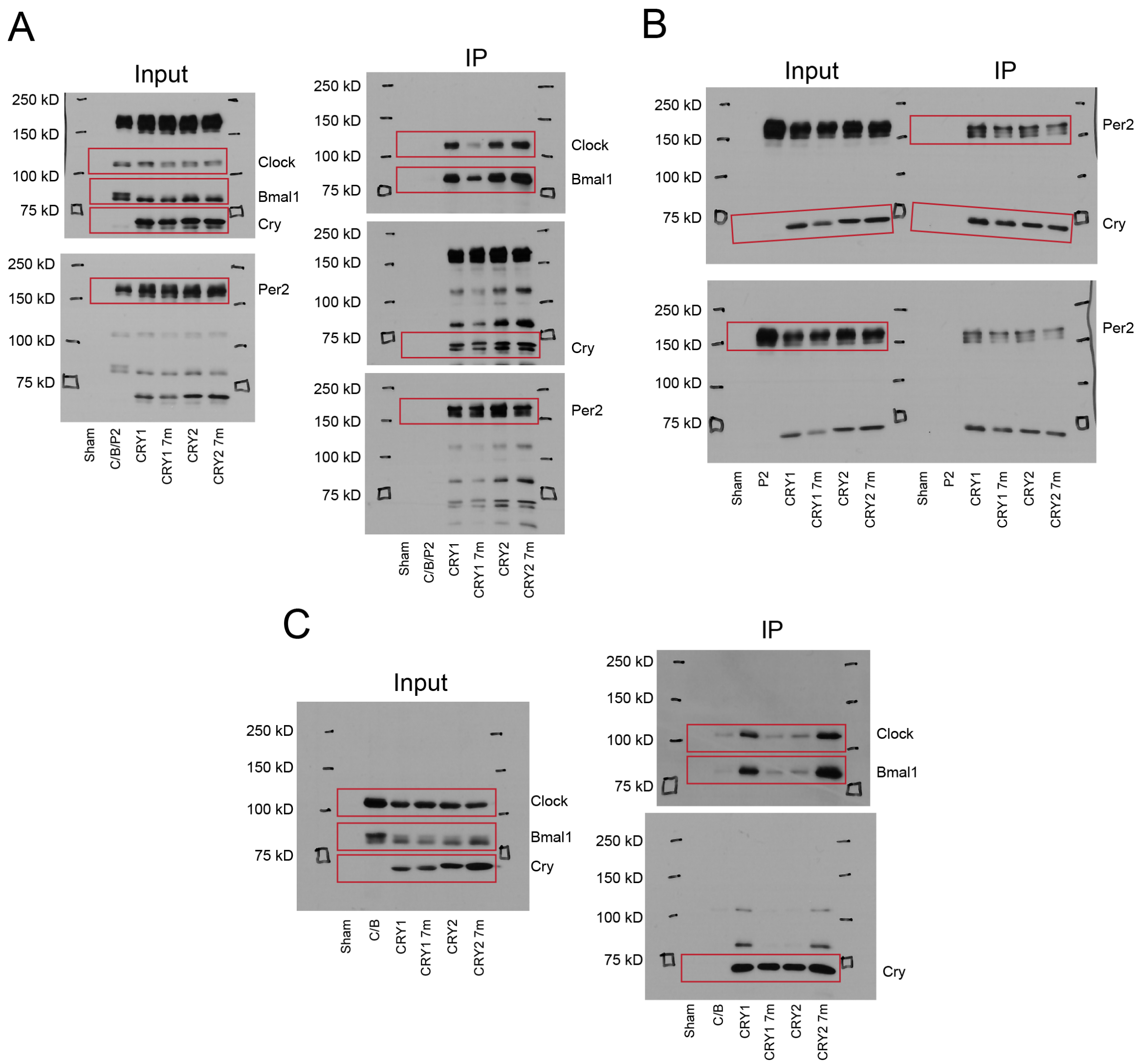

Supplementary Figure 11. Uncropped western blot images from Figure 7. (A) At left are the uncropped western blot images used for the cropped input images in Figure 7a. At right are the uncropped western blot images used for the cropped IP images in Figure 7a. (B) The left side of each image shows the uncropped western blot images used for the cropped input images in Figure 7b. The right side of each image shows the uncropped western blot images used for the cropped IP images in Figure 7b. (C) The left image shows the uncropped western blot image used for the cropped input images in Figure 7c and the right image shows the uncropped western blot image used for the cropped IP images in Figure 7c.

Supplementary Table 1. SCA sector positions. Bolded, underlined numbers represent positions that were interrogated in depth in this work.

Protein	Assigned Positions
**CRY2 residues**	26, 27, 28, 29, 30, 31, 32, 34, 35, 36, 37, 38, 39, 42, 49, 51, 53, 54, 55, **56**, **57**, **59**, 66, 68, **69**, 71, 72, 73, 74, 76, 77, 79, 80, 84, 89, 91, 93, 96, 98, 111, 114, 115, 116, 117, 118, 119, 120, **121**, 122, **123**, **124**, 127, 128, 131, 135, 140, 145, 147, 148, 149, 150, 151, 152, 153, 154, 156, 158, 161, 163, 164, 165, 166, 167, 168, 169, 170, 171, 172, 174, 187, 220, 228, 230, 231, 232, 235, 238, 239, 242, 258, 259, 260, 261, 265, 266, 267, 268, 269, 270, 271, 272, 273, 274, **275**, 276, 277, 278, 279, 280, 281, 282, 283, 284, 304, 305, 306, 307, 308, 309, 310, 311, 312, 313, 314, 316, 317, 318, 320, 321, 322, 329, 330, 333, 334, 336, 338, 345, 347, 348, 349, 351, 353, 354, 355, 356, 357, 358, 359, 360, 361, 362, 363, 364, 365, 366, 368, 369, 370, 371, 372, 373, 374, 375, 376, 377, 378, 379, 380, 381, 382, 383, 384, 385, 387, 388, 389, 390, 391, 392, 393, 395, **396**, 398, 399, **400**, 401, 402, 403, 404, 405, 406, 407, **408**, 409, 410, 411, 412, 413, 414, 415, 416, 417, 418, 419, 420, 421, 422, 423, 424, 425, 426, 427, 428, 429, 430, 431, 432, 433, 435, 436, 437, 438, 439, 440, 441, 442, 444, 446, 447, 448, 450, 451, 452, 453, 454, 457, 458, 462, 463, 465, 466, 473, 484, 485, 487, 488, 489, 490, 491, 495, 497, 498, 499, 502
**Homologous CRY1 residues**	8, 9, 10, 11, 12, 13, 14, 16, 17, 18, 19, 20, 21, 24, 31, 33, 35, 36, 37, **38**, **39**, **41**, 48, 50, **51**, 53, 54, 55, 56, 58, 59, 61, 62, 66, 71, 73, 75, 78, 80, 93, 96, 97, 98, 99, 100, 101, 102, **103**, 104, **105**, **106**, 109, 110, 113, 117, 122, 127, 129, 130, 131, 132, 133, 134, 135, 136, 138, 140, 143, 145, 146, 147, 148, 149, 150, 151, 152, 153, 154, 156, 169, 202, 210, 212, 213, 214, 217, 220, 221, 224, 240, 241, 242, 243, 247, 248, 249, 250, 251, 252, 253, 254, 255, 256, **257**, 258, 259, 260, 261, 262, 263, 264, 265, 266, 286, 287, 288, 289, 290, 291, 292, 293, 294, 295, 296, 298, 299, 300, 302, 303, 304, 311, 312, 315, 316, 318, 320, 327, 329, 330, 331, 333, 335, 336, 337, 338, 339, 340, 341, 342, 343, 344, 345, 346, 347, 348, 350, 351, 352, 353, 354, 355, 356, 357, 358, 359, 360, 361, 362, 363, 364, 365, 366, 367, 369, 370, 371, 372, 373, 374, 375, 377, **378**, 380, 381, **382**, 383, 384, 385, 386, 387, 388, 389, **390**, 391, 392, 393, 394, 395, 396, 397, 398, 399, 400, 401, 402, 403, 404, 405, 406, 407, 408, 409, 410, 411, 412, 413, 414, 415, 417, 418, 419, 420, 421, 422, 423, 424, 426, 428, 429, 430, 432, 433, 434, 435, 436, 439, 440, 444, 445, 447, 448, 455, 466, 467, 469, 470, 471, 472, 473, 477, 479, 480, 481, 484

Supplementary table 2. Primers

Primers for Agilent Site-Directed Mutagenesis

Mutation	Forward Primer (3’-->5’)	Reverse Primer (3’-->5’)
Cry1 E103K	ATCACTAAACTCTCAATTGA GTATGATTCTAAGCCT	ATCTCGTTCCTTCCCAAAAG GCTTAGAATC
Cry1 G106R	GTATGATTCTGAGCCTTTTA GGAAGGAACGAGATGCAGC	GCTGCATCTCGTTCCTTCCT AAAAGGCTCAGAATCATAC
Cry1 K107E	GAGTATGATTCTGAGCCTTT TGGGGAGGAA	GATAGCTGCATCTCGTTCCT CCCCAA
Cry1 F257A	AGTCCTTATCTCCGCGCTGG TTGTTTATC	AGATAAGGACTGAGTCCAGT TGGGC

Primers for NEB Site-Directed Mutagenesis

Mutation	Forward Primer (3’-->5’)	Reverse Primer (3’-->5’)
Cry1 D38A	TATATCCTCGCCCCCTG GTTCG	GACGCAGCGGATGGTG TC
Cry1 P39G	TATCCTCGACGGCTGGT TCGCCGG	TAGACGCAGCGGATGG TG
Cry1 F41S	GACCCCTGGTCCGCCG GCTCT	GAGGATATAGACGCAGC GGATGGTG
Cry1 G43A/N46S	TCCAGCGTGGGCATCAA CAGGTGG	AGAGGCGGCGAACCAG GGGTCGA
Cry1 R51A	GGGCATCAACGCGTGG CGATTTTTGCTTCAG	ACGTTGGAAGAGCCGG CG
Cry1 F105A	GAGATGCAGCTATCAAG AAG	GTTCCTTCCCAGCAGGC TCAG
Cry1 G106W	TGAGCCTTTTTGGAAGG AACG	GAATCATACTCAATTGA GAGTTTAG
Cry1 E108K	TTTTGGGAAGAAACGAG ATGC	GGCTCAGAATCATACTC AATTG
Cry1 E376S	CAGCTGGGAATCAGGG ATGAAGGTC	ATCCACAGGTCACCACG A
Cry1 M378V	GGAAGAAGGGGTGAAG GTCTTTG	CAGCTGATCCACAGGTC AC
Cry1 K379R	GAAGGGATGAGGGTCTT TGAAG	TTCCCAGCTGATCCACA G
Cry1 E382A	AAGGTCTTTGCAGAGTT ACTGCTTG	CATCCCTTCTTCCCAGC TG
Cry1 E382D	AGGTCTTTGATGAGTTA CTGCTTG	TCATCCCTTCTTCCCAG C
Cry1 W390F	GATGCAGATTTCAGCAT AAATGCTG	AAGCAGTAACTCTTCAA AGAC

Cry1 M378V/K379R/E382D (3m)	CTTTGATGAGTTACTGCTTGATGCAG	ACCCTCACCCCTTCTTCCCAGCTGATC
Cry1 E376S/M378V/K379R/E382D/W390F (5m)	GTTACTGCTTGATGCAGATTTTAGCATAAATGCTGGAAGTTG	TCATCAAAGACCCTCACCCCTGATTCCCAGCTGATCCACAG
Cry1 S588D	GAAGCGTCCTGATCAGGAAGAGGATGCCCAG	CCACTGCTGAGGCCGGTG
Cry2 A61G/S64N (2m)	TCGAATGTGGGCATCAACCGATGGAG	GGAGCCCGCGAACCACGGGTCGA
Cry2 V396M/R397K/D400E (3m)	ATTTGAAGAGCTGCTCCTGGATGCC	ACCTTCATCCCGCTCTCCCAGCTGAC
Cry2 S394E/V396M/R397K/D400E/F406W (5m)	GCTGCTCCTGGATGCCGATTGGAGTGTGAATGCAGGCAGC	TCTTCAAATACCTTCATCCCTTCCTCCCAGCTGACCCAGAG
pMU2-P(Cry1)-(intron336)-mCry1-Myc: Addition of Myc tag to C-term in rescue vector	AGCGAAGAAGATCTGTGAATCTATGTCGGGTGCG	AATCAGTTTCTGTTCGTTACTGCTCTGCCGCTG
pMU2-P(Cry1)-(intron336)-Myc: Deletion of mCry1 coding sequence from Myc-tagged rescue vector	GAACAGAAACTGATTAGCG	AATACCCATAATAGCTGTTTG
pCMV-Tag3C-Myc-dLuc: Deletion of mCry1 coding sequence from pCMV-Tag3C-Myc-mCry1-dLuc vector	ATGGAAGACGCCAAAAAC	TTGATATCGAATTCCTGCAG
CerC/VenC-Clock 1-395: Deletion of C-terminal residues of mClock after aa 395 in pEGFP-C1 vector	TGATCATAATCAGCCATACC	AAGAGACTCTTCAATGCC
CerC/VenC-Clock 89-395: Deletion of N-terminal residues of mClock (1-395) from aa 2-88 in pEGFP-C1 vector	CAGTCAGATGCTAGTGAGATTCGACAG	CATGCCCGCGGTACCGTC

Primers for Megaprimer Cloning

Final Vector	Primary PCR Vector	Forward Primer (3’-->5’)	Reverse Primer (3’-->5’)	Secondary PCR Vector
pMU2-P(*Cry1*)-(intron336)-mCry2-Myc	pCMV-Tag3C-Myc-mCry2	CTAGATGGCAAACAGCTATTATGGGTATTATGGCGGCGGCTGCTGTGGTGGCAGCGACG	CAGATCTTCTTCGCTAATCAGTTTCTGTTCGGAGTCCTTGCTTGCTGGCTCTTGGGTAGG	pMU2-P(Cry1)-(intron336)-Myc
pCMV-Tag3C-Myc-mCry1-dLuc	pGL3-P(Per2)-dLuc	GGCCCCAAAGTCCAGCGGCAGAGCAGTAACATGGAAGACGCCAAAAACATAAAGAAAGGC	CTTAATTAATTAAGGTACCGGGCCCCCCCTCGAGTTACACGGCGATCTTTCCGCCCTTCTTGGC	pCMV-Tag3C-Myc-mCry1
pCMV-Tag3C-Myc-mCry2-dLuc	pGL3-P(Per2)-dLuc	CCTACCCAAGAGCCAGCAAGCAAGGACTCCATGGAAGACGCCAAAAACATAAAGAAAGGC	AATTAAGGTACCGGGCCCCCCCTCGAGTCATTACACGGCGATCTTTCCGCCCTTCTTGGC	pCMV-Tag3C-Myc-mCry2

Gibson Assembly Primers

Target Vector for linearization	Forward Primer (3’-->5’) for linearization	Reverse Primer (3’-->5’) for linearization
pMU2-P(*Cry1*)-(intron336)-mCry1-Myc	GAACAGAAACTGATTAGCGAAGAAGATCTG	TAGCCCTCTGTACCGGGAAAG
pMU2-P(*Cry1*)-(intron336)-mCry1 7m-Myc	GAACAGAAACTGATTAGCGAAGAAGATCTG	TAGCCCTCTGTACCGGGAAAG
pMU2-P(*Cry1*)-(intron336)-mCry2-Myc	GAACAGAAACTGATTAGCGAAG	GAGTCCCCGGTATCTCGAC
pMU2-P(*Cry1*)-(intron336)-mCry2 7m-Myc	GAACAGAAACTGATTAGCGAAG	GAGTCCCCGGTATCTCGAC

Insert PCR Starting Vector	Forward Primer (3’-->5’) for PCR Insert	Reverse Primer (3’-->5’) for PCR Insert
pMU2-P(*Cry1*)-(intron336)-mCry1-Myc	TGTCGAGATACCGGGGACTCGGTCTTCTCGCCTCGGTC	TCGCTAATCAGTTTCTGTTCGTTACTGCTCTGCCGCTG
pMU2-P(*Cry1*)-	TTTCCCGGTACAGAGGGC	TCGCTAATCAGTTTCTGTT

(intron336)-mCry2-Myc	TATGTCTATTGGCATCTGTCCC	CGGAGTCCTTGCTTGCTGG

Supplementary Table 3. Key Resources

Reagent or Resource	Source	Identifier
**Antibodies**		
EZview Red anti-c-Myc Affinity Gel (Rabbit polyclonal anti-c-Myc)	Sigma-Aldrich	E6654
Mouse monoclonal anti-Myc	Cell Signaling	2276S
Mouse monoclonal anti-V5	Thermo Fisher Scientific	R690-25
Mouse monoclonal anti-FLAG M2-Peroxidase	Sigma-Aldrich	A8592
Anti-Mouse IgG, HRP-linked Secondary	Cell Signaling	7076S
**Chemicals, Peptides, and Recombinant Proteins**		
PfuUltra II Fusion HS DNA polymerase	Agilent Technologies	600670
D-Luciferin Firefly, sodium salt monohydrate	Biosynth	L8240
Powdered DMEM without phenol red	Corning	90-013-PB
DpnI	New England Biolabs	R0176
Quick Ligase	New England Biolabs	M2200
T4 Polynucleotide Kinase	New England Biolabs	M0201
FuGENE 6	Promega	E2692
Dexamethasone	Sigma-Aldrich	D4902
Forskolin	Sigma-Aldrich	F3917
Ampicillin	Sigma-Aldrich	A9518
Kanamycin	Sigma-Aldrich	K0129
Chloramphenicol	Sigma-Aldrich	C0378
Cycloheximide	Sigma-Aldrich	C4859
Protease Inhibitor Cocktail	Sigma-Aldrich	P8340
Fetal Bovine Serum	Sigma-Aldrich	F0926-500ML
Trizma Base	Sigma-Aldrich	T1503
Sodium Chloride	Sigma-Aldrich	S7653
Dithiothreitol (DTT)	Sigma-Aldrich	D9779
Triton X-100	Sigma-Aldrich	T8787
Sodium Dodecyl Sulfate	Sigma-Aldrich	L4509
Bromophenol Blue	Sigma-Aldrich	B5525
Tween 20	Sigma-Aldrich	P1379
β-mercaptoethanol	Thermo Fisher Scientific	AC125472500
Dulbecco's Modified Eagle Medium (DMEM)	Thermo Fisher Scientific	11965-092
Glycerol	Thermo Fisher Scientific	BP229-1
HEPES buffer	Thermo Fisher Scientific	15630
L-glutamine	Thermo Fisher Scientific	25030

Penicillin/Streptomycin Antibiotics	Thermo Fisher Scientific	15070-063
Sodium Bicarbonate	Thermo Fisher Scientific	25080
Sodium Pyruvate	Thermo Fisher Scientific	11360-070
**Commercial Assays**		
Clarity Western ECL Substrate	BioRad	170-5060
QuikChange II XL Site-Directed Mutagenesis Kit	Agilent Technologies	200521
Q5 Site-Directed Mutagenesis Kit	New England Biolabs	E0554S
2X Q5 PCR Master Mix	New England Biolabs	M0494S
NEBuilder HiFi DNA Assembly Master Mix	New England Biolabs	E2621
Qiaprep Spin Miniprep Kit	Qiagen	27106
QIAquick PCR Purification Kit	Qiagen	28104
**Deposited Data**		
NCBI non-redundant protein sequence database	National Center for Biotechnology Information	https://www.ncbi.nlm.nih.gov/protein
CPD Photolyase from *E. coli*	PDB: 1DNP	www.rcsb.org
CPD Photolyase from *A. nidulans*	PDB: 1QNF	www.rcsb.org
6-4 Photolyase from *A. thaliana*	PDB: 3FY4	www.rcsb.org
CPD Photolyase from *T. Thermophilus*	PDB: 2J07	www.rcsb.org
CRY from *D. melanogaster*	PDB: 4GU5	www.rcsb.org
CRY1 from *M. musculus*	PDB: 4K0R, 5T5X	www.rcsb.org
CRY2 from *M. musculus*	PDB: 4I6E, 4I6G	www.rcsb.org
CRY2 and FBXL3 complex from *M. musculus*	PDB: 4I6J	www.rcsb.org
CRY1 and PER2 Cry-binding domain from *M. musculus*	PDB: 4CT0	www.rcsb.org
CRY2 and PER2 Cry-binding domain from *M. musculus*	PDB: 4U8H	www.rcsb.org
**Cell lines**		
Cry1$^{-/-}$/*Cry2*$^{-/-}$ mouse embryonic fibroblasts	Gift from Andrew Liu and Hiroki Ueda, generated in [1]	
HEK 293A	Thermo Fisher Scientific	R70507
**Recombinant DNA**		
pMU2-P(*Cry1*)-(intron336)-mCry1-Myc	Modified from [1]	
pMU2-P(*Cry1*)-(intron336)-	Modified from [1]	

mCry2-Myc		
pCMV-tag3c-Myc-mCry1	From [2]	
pCMV-tag3c-Myc-mCry2	From [2]	
pCMV-tag3c-Myc-mCry1-dLuc	Modified from [2]	
pCMV-tag3c-Myc-mCry2-dLuc	Modified from [2]	
pGL3-P(*Per2*)-dLuc	From [3]	
p3XFLAG-CMV-10 DEST-mBmal1	Full length mBmal1 cDNA cloned into p3XFlag-CMV-10 at attB1 and attB2 sites using Gateway cloning	Backbone: Sigma-Aldrich E4401
p3XFLAG-CMV-10-mClock	Full-length mClock cDNA cloned into p3XFlag-CMV	Backbone: Sigma-Aldrich
pcDNA3.1-mPer2-V5	From [4]	
mCry1-CerN and mCry1-VenN	From [5]	
mCry2-CerN and mCry2-VenN	From [5]	
CerC-Clock (89-395)	Full-length *Clock* cDNA cloned into Sac2 and SmaI sites in CerC vector from [6]. Deletions made by site-directed mutagenesis	Backbone: pEGFP-C1 from Clontech 632470
H2B-mRFP1	From [7]	
**Software and Algorithms**		
pySCA v6.2	See [8]	https://github.com/reynoldsk/pySCA
Python 2.7.9	Python Software Foundation	https://python.org
iPython 3.0.0	See [9]	https://ipython.org
Custom scripts for SCA processing (alnFilterSeqSize.py, alnParseGI.py, alnReplaceHeaders.py, Header fixing script)	This work	Available upon request
MUSCLE	See [10]	http://drive5.com/muscle/
Promals3D	See [11]	http://prodata.swmed.edu/promals3d/promals3d.php
Protein BLAST	NCBI	https://blast.ncbi.nlm.nih.gov/Blast.cgi
GraphPad Prism Ver 7.0a Mac	GraphPad Software, Inc.	http://www.graphpad.com
CellProfiler Ver 2.1.1 Mac	See [12]	cellprofiler.org
MacPyMol Ver 1.7.0.3 Enhanced for Mac OS X	The PyMOL Molecular Graphics System,	

	Schrödinger, LLC	
ImageJ	NIH	https://imagej.nih.gov/ij
LumiCycle Analysis Ver 2.40	Actimetrics	http://actimetrics.com/downloads/lumicycle/
softWoRx Ver 6.5.1	GE Healthcare	
CLC Main Workbench 7 Ver 7.7.2	Qiagen	

Supplementary References

1. Ukai-Tadenuma, M. et al. Delay in feedback repression by cryptochrome 1 is required for circadian clock function. *Cell* **144**, 268-81 (2011).
2. McCarthy, E.V., Baggs, J.E., Geskes, J.M., Hogenesch, J.B. & Green, C.B. Generation of a novel allelic series of cryptochrome mutants via mutagenesis reveals residues involved in protein-protein interaction and CRY2-specific repression. *Mol Cell Biol* **29**, 5465-76 (2009).
3. Sato, T.K. et al. Feedback repression is required for mammalian circadian clock function. *Nat Genet* **38**, 312-9 (2006).
4. Kume, K. et al. mCRY1 and mCRY2 are essential components of the negative limb of the circadian clock feedback loop. *Cell* **98**, 193-205 (1999).
5. Yoo, S.H. et al. Competing E3 ubiquitin ligases govern circadian periodicity by degradation of CRY in nucleus and cytoplasm. *Cell* **152**, 1091-105 (2013).
6. Huang, N. et al. Crystal structure of the heterodimeric CLOCK:BMAL1 transcriptional activator complex. *Science* **337**, 189-94 (2012).
7. Li, Y., Yu, W., Liang, Y. & Zhu, X. Kinetochore dynein generates a poleward pulling force to facilitate congression and full chromosome alignment. *Cell Res* **17**, 701-12 (2007).
8. Rivoire, O., Reynolds, K.A. & Ranganathan, R. Evolution-Based Functional Decomposition of Proteins. *PLoS Comput Biol* **12**, e1004817 (2016).
9. Pérez, F. & Granger, B.E. IPython: a system for interactive scientific computing. *Computing in Science & Engineering* **9**, 21-29 (2007).
10. Edgar, R.C. MUSCLE: multiple sequence alignment with high accuracy and high throughput. *Nucleic Acids Res* **32**, 1792-7 (2004).
11. Pei, J., Kim, B.H. & Grishin, N.V. PROMALS3D: a tool for multiple protein sequence and structure alignments. *Nucleic Acids Res* **36**, 2295-300 (2008).
12. Kamentsky, L. et al. Improved structure, function and compatibility for CellProfiler: modular high-throughput image analysis software. *Bioinformatics* **27**, 1179-80 (2011).
